# A robust and stable gene selection algorithm based on graph theory and machine learning

**DOI:** 10.1186/s40246-021-00366-9

**Published:** 2021-11-09

**Authors:** Subrata Saha, Ahmed Soliman, Sanguthevar Rajasekaran

**Affiliations:** 1 Irving Medical Center, Columbia University, New York, NY 10032 USA; 2 Department of Computer Science and Engineering, University of Connecticut, Storrs, CT 06269 USA

**Keywords:** Robust and Stable Gene Selection Algorithm (RSGSA), Symmetric Uncertainty (SU), Gain ratio (GR), Support vector machine-recursive feature elimination (SVM-RFE), Linear Support Vector Machine (LSVM)

## Abstract

**Background:**

Nowadays we are observing an explosion of gene expression data with phenotypes. It enables us to accurately identify genes responsible for certain medical condition as well as classify them for drug target. Like any other phenotype data in medical domain, gene expression data with phenotypes also suffer from being a very underdetermined system. In a very large set of features but a very small sample size domain (e.g. DNA microarray, RNA-seq data, GWAS data, etc.), it is often reported that several contrasting feature subsets may yield near equally optimal results. This phenomenon is known as *instability*. Considering these facts, we have developed a robust and stable supervised gene selection algorithm to select a set of robust and stable genes having a better prediction ability from the gene expression datasets with phenotypes. Stability and robustness is ensured by class and instance level perturbations, respectively.

**Results:**

We have performed rigorous experimental evaluations using 10 real gene expression microarray datasets with phenotypes. They reveal that our algorithm outperforms the state-of-the-art algorithms with respect to stability and classification accuracy. We have also performed biological enrichment analysis based on gene ontology-biological processes (GO-BP) terms, disease ontology (DO) terms, and biological pathways.

**Conclusions:**

It is indisputable from the results of the performance evaluations that our proposed method is indeed an effective and efficient supervised gene selection algorithm.

## Background

Gene expression is a biochemical process where the genetic code preserved in a gene is decoded to form a specific protein and it decisively manifests an organism’s phenotypes (i.e., observable traits, such as presence of a disease, height, etc.). Therefore, regulation of gene expression is critical to an organism’s development. In the field of molecular biology, gene expression profiling is the measurement of the activity of thousands of genes simultaneously to spawn a global picture of cellular functions. Several transcriptomics technologies have been developed to produce necessary data to analyze and interpret. For instance, DNA microarrays measure the relative activity of previously identified target genes. Sequence based techniques, such as RNA Sequencing (RNA-seq), provide information on the sequences of genes in addition to their expression levels. RNA-seq based on next-generation sequencing (NGS) technologies enables transcriptome analyses of an entire genome at a very high level of resolution. These procedures stated above are not only very cost effective but also can be done in a laboratory environment.

As the quality of genome sequences and the methods for identifying protein-coding genes improved [1], the count of recognized protein-coding genes dropped to 19,000–20,000 [2]. However, a complete understanding of the role played by genes expressing regulatory RNAs that do not encode proteins has raised the total number of genes to at least 46,831 [3] in addition to another set of 2300 micro-RNA genes [4]. Since the number of samples varies from a few hundreds to a few thousands, gene selection is particularly challenging in a very underdetermined system as the system can have many equally important solutions. The algorithm of choice may select a number of random genes instead of the real set of discriminating genes. Moreover, traditional statistical methods [5] are designed to analyze susceptibility of genes from gene expression data with phenotype by considering only a single gene at a time. On the contrary, it is proven that multiple genes act together to give rise to many common diseases. There are numerous challenges in designing and analyzing joint effects of multiple differentially expressed genes. Nowadays with next generation sequencing methods (e.g., RNA-seq, CAGE, etc.), specific transcript expression can be identified. The total number of human transcripts with protein-coding potential is estimated to be at least 204,950 [6]. As stated earlier, the expressions of those transcripts are measured from not more than several thousands of individuals.

Although machine learning algorithms also suffer from very underdetermined systems, ensemble techniques can be employed in the context of supervised gene selection domain. In statistics and machine learning, ensemble learning is a machine learning technique where multiple learners are trained to solve the same problem. In contrast to ordinary machine learning approaches which try to learn one hypothesis from the training data, ensemble methods try to construct a set of hypotheses and combine them to use. We can obtain a better predictive performance by combining multiple learning algorithms instead of employing only one learner [7]. Specifically, generalization ability of an ensemble is usually much stronger than that of a single learner [8]. In this article, we have proposed an ensemble supervised gene selection algorithm dubbed as “Robust and Stable Gene Selection Algorithm” (RSGSA, in short) that is stable and robust with a better prediction ability compared to other state-of-the-art algorithms in this domain.

## Related works

Feature selection is the problem of identifying a subset of the most relevant features in the context of model construction. If the number of features is *n*, the total number of candidate subsets will be $2^n$. An exhaustive search strategy searches through all the $2^n$ feature subsets to find an optimal one. Clearly, this may not be feasible in practice [9]. A number of heuristic search strategies have been introduced to overcome this problem. In forward selection strategy, features are added one at a time. In backward selection the least important feature is removed based on some evaluation criterion. Random search strategy randomly adds or removes features to avoid being trapped in a local maximum.

Depending on the type of data, feature selection can be classified as supervised, semi-supervised, and unsupervised. A data instance (e.g., a patient potentially having cancer) is characterized by a number of independent variables (i.e., features), such as tumor markers. It may also have a response variable (often called a label), e.g., whether the patient has a benign or a malignant tumor. If all the data instances in the dataset have known response values, the process of feature selection is called “supervised.” Supervised feature selection techniques can be broadly classified into 3 categories: (1) wrapper, (2) filter, and (3) embedded. Next we illustrate each of the paradigms in brief.

### Wrapper

In a wrapper method the classification or prediction accuracy of an inductive learning algorithm of interest is used for evaluation of the generated subset. For each generated feature subset, wrappers evaluate its accuracy by applying the learning algorithm using the features residing in the subset. Although it is a computationally expensive procedure, wrappers can find the subsets from the feature space with a high accuracy [9]. Notable examples include but not limited to evolutionary algorithms [10], simulated annealing [11], and randomized search [12].

### Filter

Filter methods are computationally more efficient than wrapper methods. They evaluate the accuracy of a subset of features using objective criteria that can be assessed very quickly. Common objective criteria include mutual information, Pearson product-moment correlation coefficient, and the inter/intra class distance. Though filters are computationally more efficient than wrappers, generally they produce a less discriminating feature subset. Some notable methods include symmetric uncertainty (SU) [13], gain ratio (GR) [14], Kullback-Leibler divergence measure (KLD) [15], and RELIEF [16].

### Embedded

Embedded methods combine the qualities of filter and wrapper methods. It’s implemented by the learning algorithms that have their own built-in feature selection methods. Some of the most popular examples of these methods are linear support vector machine (LSVM) [17], random forest (RF) [18], least absolute shrinkage and selection operator (LASSO) [19], and ridge regression [20] having inbuilt penalization functions to reduce overfitting.

## Results

We have performed rigorous experimental evaluations to manifest the efficacy of our proposed method. They reveal that RSGSA outperforms the state-of-the-art algorithms in terms of stability and classification accuracy.

### Datasets

To demonstrate the effectiveness of our algorithm RSGSA, we have used 10 real microarray gene expression datasets (please, see Table 1). Details about the datasets can be found in [21]. It is to be noted that our algorithm works on any kind of gene expression datasets with phenotypes, such as RNA-Seq data.

**Table 1:** Datasets used for gene selection

Dataset	Name	# of Genes	# of Instances	# of classes
D1	Colon tumor	2000	60	2
D2	Central nervous system	7129	60	2
D3	ALL-AML	7129	72	2
D4	Breast cancer	24,481	97	2
D5	Ovarian cancer	15,154	253	2
D6	ALL-AML	7129	72	3
D7	ALL-AML	7129	72	4
D8	Lung cancer	12,533	181	3
D9	MLL	12,581	72	3
D10	SRBCT	2308	83	4

### Experimental setup

All the experiments were done on an Intel Westmere compute node with 12 Intel Xeon X5650 Westmere cores and 48 GB of RAM. The operating system running was Red Hat Enterprise Linux Server release 5.7 (Tikanga). The algorithm is written in standard Java programming language. Java source code is compiled and run by Java Virtual Machine (JVM) 1.8.0.

### Outcome

We have measured the competence of our proposed algorithm using several performance evaluation metrics, namely *Jaccard Index*, *Informedness*, and *Gain*. Please, see the Methods section for more details. Five feature selection algorithms, namely symmetric uncertainty (SU), gain ratio (GR), Kullback-Leibler divergence (KLD), RELIEF, and SVM-RFE were used to evaluate the performance of our algorithm (RSGSA). We have also employed random forest (RF) based attribute evaluation method but we are not reporting its consistently poor performance over all the datasets. Let *A* and *D* denote a specific algorithm and a specific dataset, respectively. For each dataset *D*, we execute each algorithm *A* $10 \times$ by randomly choosing $80\%$ samples from *D* with replacement each time. After each execution of *A*, we record the top *X* genes where $X = \{50, 100, 150, 200\}$. We then compute pair-wise Jaccard indices for each of *X* genes and take the average over all such pair-wise indices. The procedure was done for all the datasets by running each method *A*. Now consider an illustrative example. As we execute algorithm A $10\times$, we get 10 lists for each of top $x \in X$ genes. For example, we will get 10 lists for top 50 genes, 10 lists for top 100 genes, etc. We then compute pair-wise Jaccard indices for each top $x \in X$ genes in the corresponding 10 lists of genes and we have $\frac{10 \times (10-1)}{2} = 45$ such Jaccard indices. Finally, we take the average over all such Jaccard indices for each of the top $x \in X$ genes.

In a similar fashion, we compute the classification accuracy (i.e. *informedness*) by taking the top *X* genes selected by the algorithms. As stated above, the algorithm of interest *A* ran each time on a slightly different dataset by choosing 80% samples with replacement from *D*. The procedure is repeated $10\times$. So, at the end we will get 10 lists of genes from each top $x \in X$ genes. For each list of genes containing top *x* genes, we extract dataset $D'$ containing only top $x \in X$ genes and their corresponding expression values across the samples. We execute classifier LSVM on each dataset $D'$ containing only top $x \in X$ genes. After each execution of LSVM, we record classification accuracy (i.e. *informedness*) for top $x \in X$ genes where $X = \{50, 100, 150, 200\}$. We then compute classification accuracy by averaging over all such accuracy for each of the top $x \in X$ genes. The classification accuracy (i.e., *informedness*) is measured using 10-*fold* cross validation. Assume that $D'$ is a matrix where each row refers to a sample (i.e., individuals) and each column contains the gene expression value of a particular gene from the top *X* genes. In 10-*fold* cross-validation, the original sample ($D'$) is randomly partitioned into 10 equal sized subsamples. Of the 10 subsamples, a single subsample is retained as the validation data for testing the model, and the remaining 9 subsamples are used as training data. So, the cross-validation process is then repeated 10 times in total, with each of the 10 subsamples used exactly once as the validation data. The 10 results (i.e. *informedness*) can then be averaged to produce a single estimation. Next we discuss the performance evaluations in detail.

#### Gene selection for binary classes

**Fig. 1:**
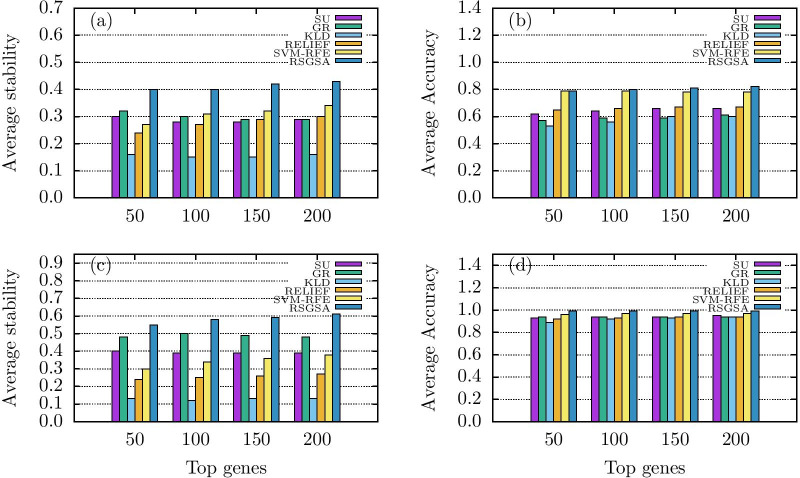
Performance evaluations **a** average *stability* for binary class **b** Average *accuracy* for binary class **c** average *stability* for multi class **d** average *accuracy* for multi class

**Fig. 2:**
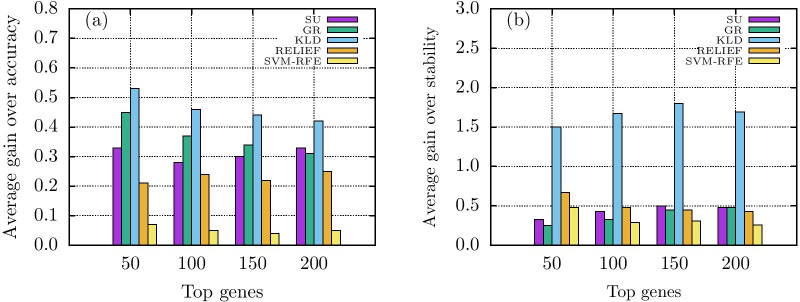
Performance evaluations of RSGSA over other notable algorithms. **a** Average gain over informedness **b** Average gain over stability

Let us first consider the performance evaluations on binary datasets (D1-D5). In terms of Jaccard index RSGSA outperforms the other algorithms in all the datasets excepting D5. Likewise, RSGSA shows a better classification accuracy over the other algorithms in all the datasets excepting D4. If we take an average over all the datasets D1-D5, RSGSA outplays the other algorithms of interest in terms of both classification stability and accuracy. Please see Table 2, Fig. 1a and b for more details. As noticeable from the performance evaluations, SVM-RFE is the closest competitor of RSGSA. Here we define improvement as the average gain over all the datasets with respect to a performance metric. RSGSA’s improvement over SVM-RFE ranges from 0.04-0.07 (i.e. 4%-7%) with respect to classification accuracy. In stability measure the average gain over SVM-RFE ranges from 0.26-0.48 (26%-48%). Please, see Fig. 2a and b for visual comparisons.

**Table 2:** Performance of two-class gene selection

		Average jaccard indices (${JI}_{avg}$)						Average informedness (${I}_{avg}$)					
Dataset	Top genes	SU	GR	KLD	RELIEF	SVM-RFE	RSGSA	SU	GR	KLD	RELIEF	SVM-RFE	RSGSA
D1	50	0.18	0.21	0.05	0.29	0.22	**0.38**	0.63	0.62	0.52	0.67	0.75	**0.86**
	100	0.21	0.21	0.11	0.31	0.28	**0.41**	0.67	0.68	0.67	0.64	0.75	**0.85**
	150	0.23	0.23	0.14	0.32	0.31	**0.44**	0.66	0.67	0.62	0.62	0.74	**0.84**
	200	0.25	0.25	0.18	0.33	0.34	**0.47**	0.66	0.68	0.61	0.62	0.74	**0.86**
D2	50	0.04	0.06	0.02	0.05	0.14	**0.23**	0.14	0.06	0.05	0.33	0.56	**0.74**
	100	0.05	0.06	0.03	0.06	0.19	**0.25**	0.21	0.17	0.01	0.34	0.59	**0.79**
	150	0.07	0.08	0.05	0.07	0.21	**0.27**	0.19	0.27	0.11	0.38	0.63	**0.79**
	200	0.08	0.08	0.07	0.08	0.24	**0.29**	0.18	0.33	0.16	0.36	0.68	**0.84**
D3	50	0.39	0.44	0.05	0.24	0.32	**0.56**	0.92	0.90	0.81	0.93	0.96	**0.99**
	100	0.35	0.43	0.05	0.22	0.34	**0.52**	0.95	0.92	0.83	0.92	0.98	**0.99**
	150	0.33	0.43	0.07	0.22	0.35	**0.53**	0.92	0.92	0.88	0.91	0.98	**0.99**
	200	0.31	0.42	0.08	0.22	0.37	**0.53**	0.93	0.93	0.88	0.94	0.97	**0.99**
D4	50	0.04	0.09	0.01	0.03	0.12	**0.17**	0.38	0.23	0.26	0.45	**0.55**	0.47
	100	0.05	0.13	0.02	0.04	0.17	**0.19**	0.39	0.22	0.31	0.40	**0.57**	0.45
	150	0.06	0.11	0.03	0.05	0.18	**0.21**	0.38	0.20	0.25	0.44	**0.61**	0.50
	200	0.07	0.11	0.04	0.06	0.20	**0.22**	0.40	0.25	0.29	0.43	**0.59**	0.52
D5	50	**0.84**	0.78	0.66	0.61	0.56	0.65	0.99	0.99	0.99	0.99	**1.00**	**1.00**
	100	**0.73**	0.65	0.54	**0.73**	0.56	0.63	**1.00**	**1.00**	**1.00**	0.99	**1.00**	**1.00**
	150	0.73	0.61	0.48	**0.81**	0.55	0.64	**1.00**	**1.00**	**1.00**	**1.00**	**1.00**	**1.00**
	200	0.74	0.60	0.44	**0.81**	0.56	0.65	**1.00**	**1.00**	0.99	**1.00**	**1.00**	**1.00**
Average	50	0.30	0.32	0.16	0.24	0.27	**0.40**	0.61	0.56	0.53	0.67	0.76	**0.81**
	100	0.28	0.30	0.15	0.27	0.31	**0.40**	0.64	0.60	0.56	0.66	0.78	**0.82**
	150	0.28	0.29	0.15	0.29	0.32	**0.42**	0.63	0.61	0.57	0.67	0.79	**0.82**
	200	0.29	0.29	0.16	0.30	0.34	**0.43**	0.63	0.64	0.59	0.67	0.80	**0.84**
Average RSGSA gain	50	0.33	0.25	**1.50**	0.67	0.48		0.33	0.45	**0.53**	0.21	0.07	
	100	0.43	0.33	**1.67**	0.48	0.29		0.28	0.37	**0.46**	0.24	0.05	
	150	0.50	0.45	**1.80**	0.45	0.31		0.30	0.34	**0.44**	0.22	0.04	
	200	0.48	0.48	**1.69**	0.43	0.26		0.33	0.31	**0.42**	0.25	0.05	

The best performance metric value among the algorithms on each dataset is highlighted in bold typeface

**Fig. 3:**
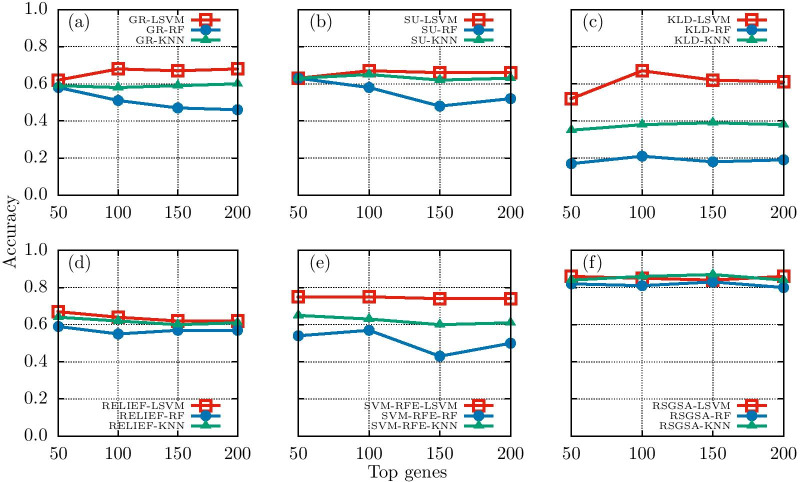
Classification accuracy of selected genes by employing LSVM, RF, and KNN classifiers for **a** GR algorithm **b** SU algorithm **c** KLD algorithm **d** RELIEF algorithm **e** SVM-RFE algorithm **f** RSGSA algorithm

To demonstrate the stability of accuracy across a set of classifiers (e.g., linear SVM (LSVM), random forest (RF), and *k*-nearest neighborhood (KNN)), we considered D1 dataset and computed classification accuracy over the top *X* genes using 10-*fold* cross validation. It is evident from Fig. 3 that all the classifiers perform equally well with respect to RSGSA’s selected top *X* genes. It further demonstrates the efficacy of our proposed method. Please see Additional file 1: Table 5 for evaluation details on all the 2-class datasets (i.e. D1-D5).

#### Gene selection for multi classes

Let us now consider the performance evaluations on multi-class datasets (D6-D10). RSGSA outperforms all the algorithms of interest in terms of both classification accuracy and stability for every dataset except for D8. On D8 dataset GR outperforms all the other algorithms including RSGSA in terms of stability. Please, see Table 3, Fig. 1c, d for more details. RSGSA’s improvement over SVM-RFE ranges from 0.02-0.03 (i.e. 2%-3%) with respect to classification accuracy. In terms of stability, the improvement over GR ranges from 0.15-0.27 (i.e. 15%-27%). Note that the classification accuracy (i.e., *informedness*) is measured by LSVM using 10-*fold* cross validation as stated above. We also computed classification accuracy based on RF and KNN classifiers. Please see Additional file 1: Table 6 for evaluation details on all the multi-class datasets (i.e., D6-D10).

**Table 3:** Performance of multi-class gene selection

		Average Jaccard indices (${JI}_{avg}$)						Average informedness (${I}_{avg}$)					
Dataset	Top genes	SU	GR	KLD	RELIEF	SVM-RFE	RSGSA	SU	GR	KLD	RELIEF	SVM-RFE	RSGSA
D6	50	0.34	0.46	0.10	0.30	0.30	**0.52**	0.93	0.94	0.87	0.94	0.96	**0.98**
	100	0.32	0.46	0.08	0.27	0.36	**0.58**	0.94	0.94	0.90	0.95	0.96	**0.99**
	150	0.31	0.43	0.08	0.27	0.36	**0.61**	0.94	0.94	0.90	0.96	0.96	**0.99**
	200	0.30	0.42	0.09	0.27	0.38	**0.62**	0.94	0.94	0.92	0.95	0.96	**0.99**
D7	50	0.39	0.49	0.12	0.17	0.25	**0.65**	0.90	0.90	0.82	0.84	0.92	**0.99**
	100	0.35	0.48	0.13	0.18	0.28	**0.62**	0.90	0.91	0.89	0.86	0.94	**0.99**
	150	0.34	0.45	0.13	0.18	0.31	**0.64**	0.90	0.91	0.90	0.87	0.95	**0.99**
	200	0.33	0.44	0.12	0.18	0.32	**0.68**	0.90	0.91	0.90	0.88	0.94	**0.99**
D8	50	0.60	**0.61**	0.19	0.20	0.22	0.50	0.89	0.91	0.89	0.90	0.95	**1.00**
	100	0.60	**0.69**	0.17	0.24	0.28	0.54	0.91	0.91	0.91	0.91	0.96	**1.00**
	150	0.60	**0.70**	0.17	0.26	0.31	0.57	0.92	0.91	0.93	0.93	0.96	**1.00**
	200	0.59	**0.68**	0.16	0.28	0.34	0.61	0.93	0.92	0.93	0.93	0.96	**1.00**
D9	50	0.34	0.36	0.10	0.24	0.29	**0.48**	0.92	0.93	0.91	0.93	**0.98**	0.97
	100	0.36	0.36	0.07	0.27	0.33	**0.50**	0.94	0.94	0.93	0.94	**0.98**	**0.98**
	150	0.36	0.37	0.07	0.27	0.35	**0.48**	0.95	0.95	0.93	0.94	**0.98**	**0.98**
	200	0.36	0.37	0.07	0.27	0.36	**0.47**	0.96	0.95	0.93	0.94	0.98	**0.99**
D10	50	0.32	0.50	0.16	0.27	0.42	**0.61**	0.99	**1.00**	0.98	0.98	0.99	**1.00**
	100	0.34	0.49	0.16	0.31	0.47	**0.64**	**1.00**	**1.00**	0.99	**1.00**	0.99	**1.00**
	150	0.36	0.49	0.18	0.34	0.48	**0.66**	**1.00**	**1.00**	**1.00**	**1.00**	**1.00**	**1.00**
	200	0.38	0.49	0.19	0.36	0.51	**0.68**	**1.00**	**1.00**	**1.00**	**1.00**	**1.00**	**1.00**
Average	50	0.40	0.48	0.13	0.24	0.30	**0.55**	0.93	0.94	0.89	0.92	0.96	**0.99**
	100	0.39	0.50	0.12	0.25	0.34	**0.58**	0.94	0.94	0.92	0.93	0.97	**0.99**
	150	0.39	0.49	0.13	0.26	0.36	**0.59**	0.94	0.94	0.93	0.94	0.97	**0.99**
	200	0.39	0.48	0.13	0.27	0.38	**0.61**	0.95	0.94	0.94	0.94	0.97	**0.99**
Average RSGSA Gain	50	0.38	0.15	**3.23**	1.29	0.83		0.06	0.05	**0.11**	0.08	0.03	
	100	0.49	0.16	**3.83**	1.32	0.71		0.05	0.05	**0.08**	0.06	0.02	
	150	0.51	0.20	**3.54**	1.27	0.64		0.05	0.05	**0.06**	0.05	0.02	
	200	0.56	0.27	**3.69**	1.26	0.61		0.04	**0.05**	**0.05**	**0.05**	0.02	

The best performance metric value among the algorithms on each dataset is highlighted in bold typeface

## Discussion

### Stability and robustness

Ensuring stability and robustness mitigates 3 key issues dominating in supervised feature selection domain: (1) In a very underdetermined system where we have few a hundreds to thousands of samples with thousands to millions of features (e.g., DNA microarray, RNA-seq data, or GWAS data), it is often found that contrasting feature subsets of similar size may yield equally identical results (such as comparable classification accuracy measured by SVMs, RFs, or Neural Networks) [22]. Aggregating a set of feature selection algorithms can aid to reduce the risk of erroneously favoring an unstable subset of features; (2) Different feature selection methods may fall in local optima in the space of feature subsets. Therefore, individually each of the algorithms can produce unstable results. Ensembling multiple feature selection techniques may yield a better approximation to the optimal subset or ranking of features; (3) Finally, the hypothesis space searched by an algorithm alone might not contain the true target function. By acting together in a concerted way, a set of feature selectors can produce a good approximation over the hypothesis space.

As noted in the Methods section, we attempt to make our learning model stable by introducing a small amount of random “noise” in the training examples, e.g., in each recursive step of SSVM-RFE, each linear SVM from the set of ensembles is trained with slightly different examples by randomly flipping class labels of a few examples. Like any other learning algorithm, SVM is also very sensitive to the training examples. As a consequence, each SVM will produce a slightly different hyperplane and we will end up with different values in the weight associated with each gene. The claim is that if a gene is stable (i.e., discriminating), it will remain stable within the small “noise” bound. At the end, SSVM-RFE assigns ranks to every gene in the dataset. The lower the rank of a particular gene, the higher will be its importance. To ensure robustness we bootstrap the training examples multiple times, i.e., we randomly select a subset of the training examples from the entire set of training examples with replacement multiple times. For each bootstrapped samples, we execute SSVM-REF and get ranks based on their importance for all the genes. Finally, we aggregate gene ranks produced by each SSVM-RFE by using Equation 3.

**Fig. 4:**
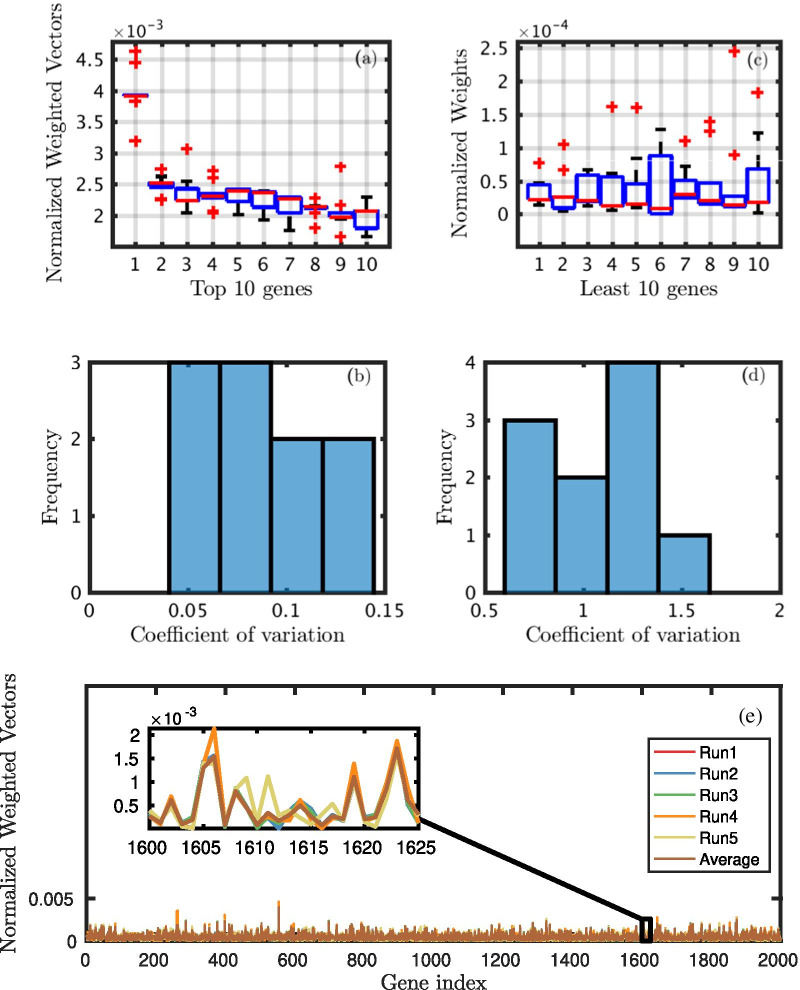
Outcome of a recursive stage of SSVM-RFE **a** Weights of top 10 genes **b** CV of top 10 genes **c** Weights of least 10 genes **d** CV of least 10 genes **e** Weights of all genes produced by 5 LSVM and their average in a recursive stage

To demonstrate the effectiveness of RSGSA in terms of *stability*, we show the outcome on D1 dataset in Fig. 4 by ensembling 5 linear SVMs in one recursive stage of SSVM-RFE. (Please note that we have used 10 LSVMs in our experiments; we have shown the output of the 5 LSVMs for clarity in Fig. 4e). One of the notable statistical measures of *stability* is the coefficient of variation (CV). It is a standardized measure of dispersion of a probability distribution or frequency distribution. CV is defined as the ratio of the standard deviation $\sigma$ to the mean $\mu$ [23]: $c_{v}= \frac{\sigma }{\mu }$. It is generally expressed as a percentage. The higher the coefficient of variation, the greater will be the level of dispersion around the mean. Therefore, the lower the value of the coefficient of variation, the more precise will be the estimate. Consider 10 most important and 10 least important genes according to their weight distribution coming from 10 linear SVMs. CVs of 10 most important genes vary from 4% to 15% (please see, Fig. 4b). Boxplots in Fig. 4a also support the evidence. On the contrary, for the 10 least important genes, CV varies from 60% to 170% (please see, Fig. 4d). Boxplots in Fig. 4c also support the findings. Figure 4e shows the weight of every gene in D1 dataset produced by 5 linear SVMs in the first recursive stage of SSVM-RFE. Please note that we randomly introduced “noise” by flipping 3% of the class labels before executing each linear SVM.

### Biological significance

We have enlisted the top 10 most important genes in Table 4. They have been selected from D1 dataset by our proposed algorithm RSGSA. Next we briefly discuss our findings considering the top 3 genes. T-cell-specific transcription factor 7 (TCF7) is directly associated with colorectal cancer [24]. The second one, TPM4, is associated with clinical progression in colon cancer patients and acts as a tumor suppressor in colon cancer cells [https://www.ncbi.nlm.nih.gov/gene/7171]. Finally, ACTB is closely associated with a variety of cancers and accumulating evidence indicates that ACTB is de-regulated in liver, melanoma, renal, colorectal, gastric, pancreatic, esophageal, lung, breast, prostate, ovarian cancers, leukemia and lymphoma [25].

**Table 4:** Top 10 genes selected by RSGSA from D1 dataset

Rank	Probe ID	Gene symbol	Full name
0	X59871	TCF7	Transcription factor 7
1	X05276	TPM4	Tropomyosin 4
2	X63432	ACTB	Actin beta
3	J05032	DARS1	aspartyl-tRNA synthetase 1
4	D26535	DLST	Dihydrolipoamide S-succinyltransferase
5	H68220	FAU	FAU ubiquitin like and ribosomal protein S30 fusion
6	T97199	ITGB4	Integrin subunit beta 4
7	T56244	PSMB2	Proteasome subunit beta 2
8	R16255	PPP3CB	Protein phosphatase 3 catalytic subunit beta
9	T70063	EIF4G2	Eukaryotic translation initiation factor 4 gamma 2

Now consider the biological significance of the top 100 genes selected from colon tumor dataset (D1) by our proposed method RSGSA. They can be found at Additional file 1: Table 4. The enrichment analysis is based on three dimensions, e.g., gene ontology-biological processes (GO-BP) terms, disease ontology (DO) terms, and biological pathways. Please, note that GO-BP and DO analyses were performed using “clusterProfiler” [26]. Biological pathways are extracted from “ConsensusPathDB-human” (CPDB, in short) [http://cpdb.molgen.mpg.de/]. Next we briefly illustrate each of the enrichment analyses.

#### Gene ontology-biological processes (GO-BP)

One of the main uses of the GO terms is to perform enrichment analysis on a given set of genes. For instance, an enrichment analysis will find which GO terms are over-represented (or under-represented) using annotations for that set of genes. We have performed enrichment analysis on the set of 100 genes as noted above based on GO-BP terms and retained 104 Benjamini-Hochberg corrected (adjusted $p < 0.05$) GO-BP terms. Top 10 enriched GO-BP terms can be found in Table 5. Please, see Additional file 1: Table 1 for details about those terms. Most of the terms are associated with colon cancer. For example, the authors in [27] have described CRC and Renal Cell Carcinoma (RCC) as concomitant malignancies in their study of patients carrying both types of cancer. Patients with Retinoblastoma are also shown to have a high risk of getting colon cancer over time [28].

**Table 5:** Top 10 enriched (GO-BP) terms

ID	Description	*p* value	*p* adjusted
GO:0001503	Ossification	2.89E-06	0.007458585
GO:0016051	Carbohydrate biosynthetic process	9.61E-06	0.012383589
GO:0006352	DNA-templated transcription, initiation	3.30E-05	0.018847646
GO:0006367	Transcription initiation from RNA polymerase II promoter	3.96E-05	0.018847646
GO:0035270	Endocrine system development	4.28E-05	0.018847646
GO:0070371	ERK1 and ERK2 cascade	5.02E-05	0.018847646
GO:0002683	Negative regulation of immune system process	6.27E-05	0.018847646
GO:0006006	Glucose metabolic process	7.11E-05	0.018847646
GO:0010038	Response to metal ion	7.30E-05	0.018847646
GO:0031018	Endocrine pancreas development	7.31E-05	0.018847646

#### Disease ontology (DO)

Like GO, the disease ontology (DO) is a formal ontology of human disease. We have performed enrichment analysis on the set of top 100 genes as noted above based on DO terms and retained 17 Benjamini-Hochberg corrected (with an adjusted $p < 0.05$) DO terms. Top 10 enriched DO terms can be found in Table 6. Please see Additional file 1: Table 2 for details about those terms. Almost all of the retained enriched DO terms are associated with colon cancer. For an instance, although a rare case, it has been reported that a 76-year-old woman has a colon cancer with ossification [29]. Relation between colorectal cancer and ossification is also demonstrated in [43] and [44]. As another example, it has been shown that colon cancer progression has been impaired via inactivating the Wnt pathway [30].

**Table 6:** Top 10 enriched disease ontology (DO) terms

ID	Description	*p* value	*p* adjusted
DOID:3996	Urinary system cancer	0.000161338	0.031918678
DOID:4450	Renal cell carcinoma	0.00027404	0.031918678
DOID:0060116	Sensory system cancer	0.000278766	0.031918678
DOID:2174	Ocular cancer	0.000278766	0.031918678
DOID:4451	Renal carcinoma	0.000670596	0.03438733
DOID:768	Retinoblastoma	0.000674808	0.03438733
DOID:771	Retinal cell cancer	0.000674808	0.03438733
DOID:4645	Retinal cancer	0.000738581	0.03438733
DOID:14067	Plasmodium falciparum malaria	0.000771616	0.03438733
DOID:2377	Multiple sclerosis	0.000903255	0.03438733

#### Biological pathways

We have also performed biological pathway analysis and retained 23 Bonferroni adjusted (with $p < 0.05$) enriched pathways. Almost all of the retained pathways are associated with colon cancer. Top 10 enriched biological pathways can be found in Table 7. Please see Additional file 1: Table 3 for details about those pathways. Now we illustrate some of the pathways in detail. Interleukins are a group of cytokines that contribute to growth and differentiation, cell migration, and inflammatory and anti-inflammatory responses by the immune system. In a study, authors in [31] examined genetic variation in genes from various anti-inflammatory and pro-inflammatory interleukins to determine association with colon and rectal cancer risk and overall survival. Data from two population-based incident studies of colon cancer (1,555 cases and 1,956 controls) and rectal cancer (754 cases and 954 controls) were utilized. After controlling for multiple comparisons, authors found that single nucleotide polymorphisms (SNPs) from four genes, IL3, IL6R, IL8, IL15, were associated with increased colon cancer risk. It has also been discovered that colorectal cancer cells express a hybrid form of $\alpha 6\beta 4$ that is never seen in normal cells [32]. The expression levels of epidermal growth factor receptors (EGFRs) vary significantly on normal and malignant colon epithelial cells [33]. Furthermore, activation of the EGFR signaling pathway was proposed as a rational target for anti-tumor drugs [34]. In addition, Human T-lymphotropic virus-I (HTLV-I) is one of the retroviruses associated with human cancer [35].

**Table 7:** Top 10 enriched biological pathways

Pathway	Source	ID	Hypergeometric *p* value
IL3	NetPath	Pathway_IL3	2.75E-06
Cellular senescence	KEGG	path:hsa04218	4.51E-06
CD4 T cell receptor signaling	INOH	None	8.12E-06
VEGF	INOH	None	1.59E-05
Alpha6Beta4Integrin	NetPath	Pathway_Alpha6Beta4Integrin	1.93E-05
TCR	NetPath	Pathway_TCR	2.14E-05
Fibroblast growth factor-1	NetPath	Pathway_Fibroblast__growth__factor-1	2.44E-05
a6b1 and a6b4 Integrin signaling	PID	a6b1_a6b4_integrin_pathway	2.59E-05
BCR	NetPath	Pathway_BCR	6.87E-05
EPO signaling	INOH	None	1.02E-04

## Conclusions

In this article, we have proposed a robust and stable supervised gene selection algorithm RSGSA based on graph theory and ensembles of linear SVMs. At the beginning, highly correlated genes are discarded by employing a novel graph theoretic algorithm. Stability of SVM-RFE is ensured by introducing a small “noise” in phenotypes. Robustness is secured by instance level perturbation (i.e., bootstrapping samples multiple times). Rigorous experimental evaluations were performed on 10 real gene expression datasets. It is evident from the results of the performance evaluations that RSGSA is indeed an effective and efficient supervised gene selection algorithm.

## Methods

Ensemble feature selection techniques might be employed in the domain of supervised gene selection to ensure *stability* and *robustness*. In this context, we define the *stability* and *robustness* as they have some clear distinction between them. In the context of gene selection algorithm, we can define *robustness* as the variation of selected genes resulting from small changes in the dataset, such as adding or removing samples from the dataset. Likewise, we can define the *stability* of gene selection algorithms as the variation in gene selection results due to adding a small amount of “noise” in the dataset. The less the variation, the more the algorithm will be robust and stable. Both *robustness* and *stability* are desirable characteristics of a gene selection algorithm, especially where the number of biomarkers is much larger than the number of samples.

Our algorithm RSGSA runs in 2 stages. At first, it removes highly correlated genes and thus, retains only approximately independent genes. These independent genes are then ranked based on their importance by cleverly utilizing a set of linear SVMs. To ensure *robustness* we bootstrapped the samples multiple times to create slightly different datasets by randomly taking samples with replacement. For each dataset we run our gene ranking algorithm. To ensure stable ranking a small amount of “noise” is introduced in the ranking algorithm by randomly flipping class labels of a few samples. To get an in-depth knowledge about ensemble techniques for feature selection, the readers are referred to [36]. To the best of our knowledge, the algorithmic steps we follow, such as removing correlated features and ensuring stability in SVM-RFE’s recursive stage are unique in this domain. Next we describe our algorithmic framework in detail.

### Algorithm 1: Removing correlated genes

Principally, correlated genes do not improve the learning model of interest. There are basically 3 main reasons to remove correlated genes from the set of given genes: (1) making the gene selection algorithm faster; (2) decreasing harmful bias; and (3) making the model simpler and explainable.

We have designed and developed a novel graph theoretic algorithm to effectively remove the correlated genes from consideration. It works as follows.

Suppose we have a set of genes *S*. A graph *G* is constructed in which there is a node for each gene $s\in S$. Two nodes $n_1$ and $n_2$ in *G* will be connected by an edge *e* if $r(n_1, n_2) \ge \lambda$ where *r* is the Pearson’s correlation coefficient and $\lambda$ is a user defined threshold. In our experiments, we set $\lambda = 0.9$. After constructing such a graph *G*, we extract all the connected components of *G*. For each connected component, we measure *eigenvector centrality* [37] of each node residing in that component. In graph theory, *eigenvector centrality* (also called *eigencentrality*) is a measure of the influence of a node in a network. Relative scores are assigned to all nodes in the network based on the concept that connections to high-scoring nodes contribute more to the score of the node in question than equal connections to low-scoring nodes. A node having a high eigenvector score demonstrates the fact that it is connected to many nodes who themselves have high eigenvector scores. Let a node *n* have the highest eigenvector score across all the connected components. We delete all the neighboring nodes of *n* and *n* from *G*. We record *n* as a *leader* of its neighbors. Since, all the neighbors of *n* are connected with *n*, they are highly correlated with *n*. So, deleting the neighbors of *n* will not only cost minimal information loss but also reduce the dimension of the gene space. The same procedure is repeated until all the nodes $n \in G$ are isolated, i.e., there is no edge *e* in *G*. We record all such nodes *n* as *leaders*. These *leaders* are then ranked based on their importance as stated below. The details of the algorithm can be found in Algorithm 1. Note that we also return all the neighbors of each *leader* after ranking. So, there is no information loss due to pruning of the genes. 
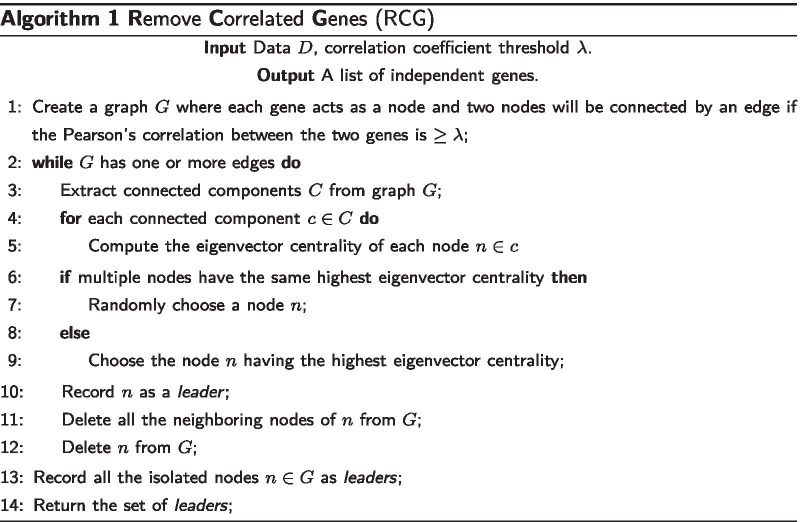


### Algorithm 2: Ranking genes

Recursive feature elimination method based on support vector machines (SVM-RFE, in short) was first proposed in [17] to rank genes based on their importance for cancer classification. It works by recursively removing one or more weak features (e.g., genes) until the specified number of features is reached. We have provided a short summary of SVM-RFE in Appendix.

In this article, we attempt to make SVM-RFE stable by introducing small changes in class labels and employing ensembles of LSVMs in each recursive step to eliminate a set of least important genes. Due to this small change, the weight associated with each gene will not be identical for the identical dataset in different runs. By averaging the weights across a set of weight vectors from LSVMs of the same configuration, we can make the weight vector stable. Since, weights are directly associated with the importance of the genes, ranking should also be stable. We call this variation of SVM-RFE as Stable SVM-RFE (SSVM-RFE, in short). Let the number of recursive steps taken by SSVM-RFE to rank a set *S* of genes of interest be *R*. Suppose in each of the *R* recursive steps we employ a set of *L* LSVMs. Each LSVM $l \in L$ is trained to build an inductive learning model by introducing small changes in class labels and we get a corresponding weight vector $w_{l}$ ($1 \le l \le |L|$). According to [17] the importance of the $i^{th}$ gene $I_{i} = (w_{l}^{i})^2$ where $w_{l}^{i}$ represents the $i^{th}$ weight component of $w_{l}^{th}$ weight vector. At the end of each recursive step, we get |*L*| weight vectors. Let the set of weight vectors obtained in the $r^{th}$ recursive step be denoted as $W_{r}$, $1\le r\le R$. Since in each run we introduce small “noise” in class labels, the weight vectors will be different from each other. To make it stable we normalize each weight vector and average each component. Let the number of genes remaining in a particular run be *n*. Each weight vector $w_{l}$ will have *n* components and is normalized as follows:

1 $$\begin{aligned} w_{l}' = \frac{w_{l}}{\sum _{i = 1}^{n}|w_{l}^{i}|} \end{aligned}$$

The $i^{th}$ component of the final weight vector $W_{r}$ for the $r^{th}$ recursive step ($1\le r\le R$) is formed as follows:

2 $$\begin{aligned} W_{r}^{i} = \sum _{l = 1}^{|L|}(w_{l}^{i})^{2} \end{aligned}$$

At the end of the $r^{th}$ recursive step, the importance of the $i^{th}$ gene is defined as $I^r_{i} = W_{r}^{i}$ and we discard one or more genes having the least scores. The above procedure is done recursively until we have the desired number of genes left. Consequently, SSVM-RFE outputs the most important genes. We claim that these ranks are stable.

To make our algorithm robust, we repeatedly run the above procedure on different bootstrapped samples. If the dataset is imbalanced, our algorithm automatically makes it balanced before bootstrapped sampling by creating synthetic samples from the minority class instead of creating copies [38]. The final ranking is done by aggregating all the rankings produced from different samples. Let the number of SSVM-RFE runs employed be *m*. Each SSVM-RFE run produces a list of stable ranks for the given set of genes *S*. Let the $i^{th}$ SSVM-RFE run provide a gene ranking $s_{i} = \{s_{i}^{1}, \ldots , s_{i}^{|S|}\}$ where $1 \le i \le m$. We can aggregate the gene rankings by taking the sums as follows:

3 $$\begin{aligned} s^{j} = \sum _{i = 1}^{m}s_{i}^{j} \end{aligned}$$

where *j* represents the $j^{th}$ gene. The pseudo code of our algorithm can be found in Algorithm 2. Please, note that the input data matrix *D* is formed using Algorithm 1. 
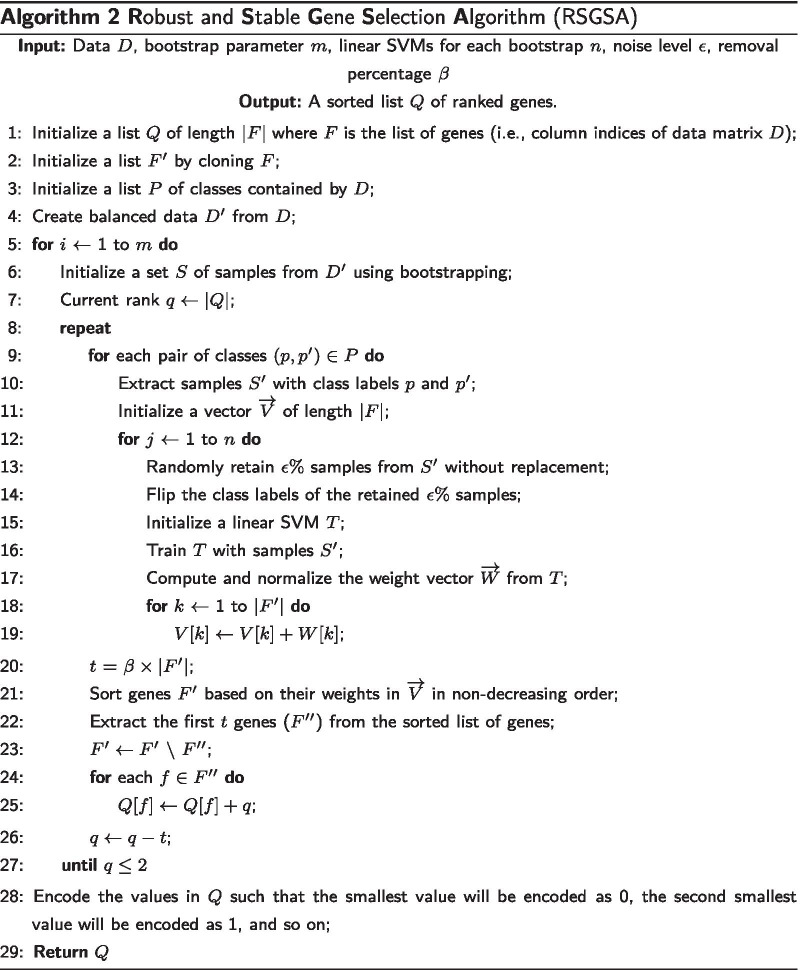


### Analysis

In this section we analyze the time complexity of the algorithm. Let *n* be the number of genes in the input. Let *e* be the number of training examples.

In Algorithm 1, we can construct the graph *G*(*V*, *E*) in time $O(n^2e)$, since the Pearson’s correlation coefficient between any two genes can be computed in *O*(*e*) time. Given a graph *G*(*V*, *E*), the problem of computing the eigencentrality for each node can be reduced to the problem of computing the eigen vectors of a $|V|\times |V|$ matrix [39]. As a result, if there are *n* genes, then their eigencentrality values can be computed in $O(n^3)$ time (since eigen vectors for a $n\times n$ matrix can be computed in $O(n^3)$ time [40, 41]).

In Algorithm 1, we start with *n* genes, find a node with the highest eigencentrality, and remove all of its neighbors and itself. We repeat the process of isolating a node and its neighbors until there are no more edges in the graph. This process is described in lines 2 to 14. In each iteration of this while loop the number of remaining genes decreases. Note that in the worst case only one edge (and two nodes) from the graph might get deleted. This means that the total time for computing the eigencentrality for the nodes across all the iterations of the while loop is $O(n^4)$. Please note that this is the worst case which does not arise in practice. In practice, our algorithm is very fast.

Thus the run time of Algorithm 1 is $O(n^2e+n^4)$.

In Algorithm 2, let *N* be the starting number of genes. For example, let *N*/2 be the target number of genes to be output. In each iteration of the repeat loop of line 8, a constant fraction of the genes with the lowest weights are eliminated. In other words, the repeat loop is executed only $R=O(1)$ times. For instance, in the first iteration we eliminate $\frac{N}{2R}$ genes; In the second iteration we eliminate another set of $\frac{N}{2R}$ genes, and so on.

In every iteration of the repeat loop we have to train a linear SVM |*L*| times. The run time of linear SVM is $O(ab\min \{a,b\})$ where *a* is the number of attributes and *b* is the number of training examples (see e.g., [42]). As a result, the time spent in one execution of the repeat loop is $O(|L|RNe\min \{N,e\})$.

The for loop of line 5 is executed *m* times. As a result, the total run time of Algorithm 2 is $O(m|L|RNe\min \{N,e\})$.

Put together, the run time of our algorithm is $O(n^2e+n^4+m|L|RNe\min \{N,e\})$. If $n>e$, this run time will be $O(n^4+m|L|Rne^2)$. On the other hand, if $e\ge n$, then the run time will be $O(n^2e+n^4+m|L|Rn^2e)$. We arrive at the following Theorem:

#### Theorem 1

The run time of RSGSA is $O(n^2e+n^4+m|L|RNe\min \{N,e\})$. If $n>e$, this run time will be $O(n^4+m|L|Rne^2)$. On the other hand, if $e\ge n$, then the run time will be $O(n^2e+n^4+m|L|Rn^2e)$.

### Evaluation metrics

We have measured the effectiveness of our proposed algorithm RSGSA using 3 different performance metrics. These metrics are defined below.

#### Jaccard index

*Stability* is measured by employing Jaccard index. It measures similarity between finite sample sets, and is defined as the size of the intersection divided by the size of the union of the sample sets: $J(A, B) = \frac{|A \cap B|}{|A \cup B|}$ where *A* and *B* are two sample sets. The Jaccard index varies from 0.0 to $\texttt {+}1.0$. The higher the value, the more similar will be the two sets.

#### Informedness

We are interested in evaluating the goodness of a classifier *C* in correctly identifying positive and negative examples from a set of examples. For instance, positive and negative examples in binary classification problem can be referred to as persons having a specific disease and healthy individuals, respectively. We refer to positive examples that are identified as positive as true positives (TP), positive examples that are identified as negative as false negatives (FN), negative examples that are identified as positive as false positives (FP), and negative examples that are identified as negative as true negatives (TN) by a classification algorithm *C*.

*Informedness* is a measure of how informed system *C* is about positives and negatives, i.e. $Informedness = \frac{TP}{TP + FN} - \frac{FP}{FP + TN}$. It can be also written as: $Informedness = sensitivity + specificity - 1$. It varies from $\texttt {-}1.0$ to $\texttt {+}1.0$. A value of $\texttt {+}1.0$ implies that *C* is fully informed about positives and negatives.

In this article *informedness* is also termed as *accuracy*, i.e., these two terms are interchangeable throughout this article. Note that *informedness* is measured using 10-*fold* cross validation.

#### Gain

Gain measures the percentage improvement over any performance metric (such as Jaccard index, informedness, etc.) achieved by RSGSA when compared to other algorithms. Let us assume the performance metric of interest of RSGSA and another algorithm of interest be $p'$ and $p''$, respectively. The gain is measured using this formulation: $Gain = \frac{p' - p''}{p''} \times 100.0 \%$.

### Additional file

**Additional file 1.** Detailed biological and performance analyses.

## Data Availability

The datasets analyzed during the current study are available in the “Microarray Dataset” repository, 10.6084/m9.figshare.9642569.v1 [46]. The implementations of our algorithms are freely available for non-commercial purposes. These implementations can be downloaded from the “RSGSA’s executable” repository, 10.6084/m9.figshare.9642581.v2 [47].

## Appendix

Support Vector Machine (SVM) is a classic supervised machine learning model [45]. SVM effectively solves both classification and regression problems. Based on a subset of training points and a proper selection of a kernel function, SVM identifies an optimum decision function. For example, Linear SVM is an SVM that uses a linear kernel function. This decision function could then be used to predict the class membership of new data points (i.e., decide the best class to which the new data point potentially belongs). Note that SVM could solve both types of classification problems, namely binary and multi-class. In multi-class classification problems, the problem is reduced to training multiple binary classifiers. Each binary classifier is trained to decide whether a new data point belongs to one particular class label or the others. Finally, the decision is based on a voting system that analyzes all the labels generated by all the binary classifiers.

At the beginning, a linear SVM (LSVM, in short) is trained on the initial set of features to build an inductive learning model. In binary classification problem this model is nothing but a vector of weights (also known as coefficient vector) describing a separating hyperplane between two classes. Each entry of the vector corresponds to the coefficient of a particular feature. The importance of each feature is directly proportional to its coefficient in the weight vector [17]. The more the weight of a feature the more will it be important. The least important features are then pruned from the current set of features. This procedure is recursively repeated on the current set of features until the desired number of most important features is reached.
