# Supplementary material for: A robust and stable gene selection algorithm based on graph theory and machine learning
Source: Hum Genomics. 2021 Nov 9;15:66. doi: 10.1186/s40246-021-00366-9 (PMC8579680; doi:10.1186/s40246-021-00366-9)
Supplement: Supplementary file 1 — Additional file 1. Detailed biological and performance analyses. [file 40246_2021_366_MOESM1_ESM.pdf]

Supplementary Materials

RSGSA: a robust and stable gene selection algorithm

Subrata Saha, Ahmed Soliman, and Sanguthevar Rajasekaran

Table 1: Gene Ontology-Biological Processes (GO-BP) Analysis

ID	Description	p-value	p-adjust
GO:0001503	ossification	0.000002893	0.007458585
GO:0016051	carbohydrate biosynthetic process	0.000009607	0.012383589
GO:0006352	DNA-templated transcription, initiation	0.000032957	0.018847646
GO:0006367	transcription initiation from RNA polymerase II promoter	0.000039637	0.018847646
GO:0035270	endocrine system development	0.000042843	0.018847646
GO:0070371	ERK1 and ERK2 cascade	0.000050224	0.018847646
GO:0002683	negative regulation of immune system process	0.000062710	0.018847646
GO:0006006	glucose metabolic process	0.000071114	0.018847646
GO:0010038	response to metal ion	0.000073007	0.018847646
GO:0031018	endocrine pancreas development	0.000073110	0.018847646
GO:0034248	regulation of cellular amide metabolic process	0.000084510	0.019301154
GO:0001649	osteoblast differentiation	0.000095972	0.019301154
GO:0016055	Wnt signaling pathway	0.00010117	0.019301154
GO:0198738	cell-cell signaling by wnt	0.000104816	0.019301154
GO:0006091	generation of precursor metabolites and energy	0.000116432	0.020010836
GO:0051591	response to cAMP	0.000128794	0.020751854
GO:0006417	regulation of translation	0.000175337	0.025394423
GO:0071900	regulation of protein serine/threonine kinase activity	0.000177308	0.025394423
GO:0070372	regulation of ERK1 and ERK2 cascade	0.000211407	0.027445823
GO:0019318	hexose metabolic process	0.000215804	0.027445823
GO:0010288	response to lead ion	0.00022357	0.027445823
GO:0002068	glandular epithelial cell development	0.000253143	0.029663758
GO:0002685	regulation of leukocyte migration	0.000279036	0.031161063
GO:0072091	regulation of stem cell proliferation	0.000290095	0.031161063
GO:0035116	embryonic hindlimb morphogenesis	0.000356691	0.036313707
GO:0033692	cellular polysaccharide biosynthetic process	0.000366236	0.036313707
GO:0006929	substrate-dependent cell migration	0.000396418	0.037120766
GO:0030278	regulation of ossification	0.000405019	0.037120766
GO:0035265	organ growth	0.00042781	0.037120766
GO:0043687	post-translational protein modification	0.00043641	0.037120766
GO:0002576	platelet degranulation	0.000446371	0.037120766
GO:0051098	regulation of binding	0.000504403	0.038032421
GO:0000271	polysaccharide biosynthetic process	0.000505829	0.038032421
GO:0051403	stress-activated MAPK cascade	0.000538654	0.038032421
GO:0005996	monosaccharide metabolic process	0.000573296	0.038032421

GO:0001914	regulation of T cell mediated cytotoxicity	0.000583461	0.038032421
GO:0031016	pancreas development	0.000588204	0.038032421
GO:0046683	response to organophosphorus	0.000608577	0.038032421
GO:0050900	leukocyte migration	0.000621255	0.038032421
GO:0030224	monocyte differentiation	0.000637629	0.038032421
GO:0071868	cellular response to monoamine stimulus	0.000637629	0.038032421
GO:0071870	cellular response to catecholamine stimulus	0.000637629	0.038032421
GO:1903131	mononuclear cell differentiation	0.000637629	0.038032421
GO:0034250	positive regulation of cellular amide metabolic process	0.000671395	0.038032421
GO:0016049	cell growth	0.000689071	0.038032421
GO:0009127	purine nucleoside monophosphate biosynthetic process	0.000693376	0.038032421
GO:0009168	purine ribonucleoside monophosphate biosynthetic process	0.000693376	0.038032421
GO:0035137	hindlimb morphogenesis	0.000755359	0.040569051
GO:0034637	cellular carbohydrate biosynthetic process	0.000780813	0.041080332
GO:2000648	positive regulation of stem cell proliferation	0.00081906	0.042230744
GO:0035264	multicellular organism growth	0.000836779	0.042298368
GO:0071867	response to monoamine	0.000886077	0.043100129
GO:0071869	response to catecholamine	0.000886077	0.043100129
GO:0014074	response to purine-containing compound	0.000943963	0.044712491
GO:0031098	stress-activated protein kinase signaling cascade	0.000953913	0.044712491
GO:0031341	regulation of cell killing	0.000972292	0.044760155
GO:0009156	ribonucleoside monophosphate biosynthetic process	0.001061049	0.045365541
GO:0035878	nail development	0.001073428	0.045365541
GO:0072584	caveolin-mediated endocytosis	0.001073428	0.045365541
GO:1902004	positive regulation of amyloid-beta formation	0.001073428	0.045365541
GO:1990535	neuron projection maintenance	0.001073428	0.045365541
GO:0048010	vascular endothelial growth factor receptor signaling pathway	0.001101831	0.045814831
GO:0032147	activation of protein kinase activity	0.001144623	0.046552893
GO:0001906	cell killing	0.001155696	0.046552893
GO:0071675	regulation of mononuclear cell migration	0.001188614	0.047142249
GO:0071496	cellular response to external stimulus	0.001250594	0.047246623
GO:0007050	cell cycle arrest	0.00128996	0.047246623
GO:0061052	negative regulation of cell growth involved in cardiac muscle cell development	0.001307723	0.047246623
GO:0001101	response to acid chemical	0.001340792	0.047246623
GO:0046364	monosaccharide biosynthetic process	0.001343908	0.047246623
GO:0007596	blood coagulation	0.001387763	0.047246623
GO:0010634	positive regulation of epithelial cell migration	0.001400754	0.047246623
GO:0031330	negative regulation of cellular catabolic process	0.001402988	0.047246623
GO:0070374	positive regulation of ERK1 and ERK2 cascade	0.001402988	0.047246623
GO:0043405	regulation of MAP kinase activity	0.001411726	0.047246623
GO:0002686	negative regulation of leukocyte migration	0.001453475	0.047246623

GO:0009124	nucleoside monophosphate biosynthetic process	0.001477318	0.047246623
GO:0048872	homeostasis of number of cells	0.00149261	0.047246623
GO:1904951	positive regulation of establishment of protein localization	0.001501824	0.047246623
GO:0044264	cellular polysaccharide metabolic process	0.001505755	0.047246623
GO:0007599	hemostasis	0.001510838	0.047246623
GO:0050817	coagulation	0.001536445	0.047246623
GO:0005978	glycogen biosynthetic process	0.001549315	0.047246623
GO:0009250	glucan biosynthetic process	0.001549315	0.047246623
GO:0006979	response to oxidative stress	0.001590515	0.047246623
GO:0090257	regulation of muscle system process	0.001618827	0.047246623
GO:0030098	lymphocyte differentiation	0.001642286	0.047246623
GO:0002067	glandular epithelial cell differentiation	0.001649028	0.047246623
GO:0007528	neuromuscular junction development	0.001649028	0.047246623
GO:0060079	excitatory postsynaptic potential	0.001742047	0.047246623
GO:0070849	response to epidermal growth factor	0.00175267	0.047246623
GO:0070482	response to oxygen levels	0.001782493	0.047246623
GO:0006164	purine nucleotide biosynthetic process	0.001823205	0.047246623
GO:0031668	cellular response to extracellular stimulus	0.001823205	0.047246623
GO:0043406	positive regulation of MAP kinase activity	0.001823205	0.047246623
GO:0042110	T cell activation	0.001830537	0.047246623
GO:0033127	regulation of histone phosphorylation	0.001842615	0.047246623
GO:0060712	spongiotrophoblast layer development	0.001842615	0.047246623
GO:0071287	cellular response to manganese ion	0.001842615	0.047246623
GO:1902993	positive regulation of amyloid precursor protein catabolic process	0.001842615	0.047246623
GO:0046390	ribose phosphate biosynthetic process	0.001859088	0.047246623
GO:0001909	leukocyte mediated cytotoxicity	0.001869339	0.047246623
GO:1905475	regulation of protein localization to membrane	0.001907239	0.047736531
GO:0001913	T cell mediated cytotoxicity	0.001971961	0.04888189

Table 2: Disease Ontology (DO) Analysis

ID	Description	p-value	p-adjust
DOID:3996	urinary system cancer	0.00016	0.03192
DOID:4450	renal cell carcinoma	0.00027	0.03192
DOID:0060116	sensory system cancer	0.00028	0.03192
DOID:2174	ocular cancer	0.00028	0.03192
DOID:4451	renal carcinoma	0.00067	0.03439
DOID:768	retinoblastoma	0.00067	0.03439
DOID:771	retinal cell cancer	0.00067	0.03439
DOID:4645	retinal cancer	0.00074	0.03439
DOID:14067	plasmodium falciparum malaria	0.00077	0.03439

**DOID:2377**	**multiple sclerosis**	**0.0009**	**0.03439**
**DOID:557**	**kidney disease**	**0.00096**	**0.03439**
**DOID:3213**	**demyelinating disease**	**0.00111**	**0.03439**
**DOID:2048**	**autoimmune hepatitis**	**0.00118**	**0.03439**
**DOID:2151**	**malignant ovarian surface epithelial-stromal neoplasm**	**0.0012**	**0.03439**
**DOID:2152**	**ovary epithelial cancer**	**0.0012**	**0.03439**
**DOID:4001**	**ovarian carcinoma**	**0.0012**	**0.03439**
**DOID:18**	**urinary system disease**	**0.00132**	**0.03551**

Table 3: Biological Pathway Analysis

**Pathway**	**Source**	**ID**	**Hypergeometric p-value**
**IL3**	**NetPath**	**Pathway_IL3**	**2.75E-06**
**Cellular senescence**	**KEGG**	**path:hsa04218**	**4.51E-06**
**CD4 T cell receptor signaling**	**INOH**	**None**	**8.12E-06**
**VEGF**	**INOH**	**None**	**1.59E-05**
**Alpha6Beta4Integrin**	**NetPath**	**Pathway_Alpha6Beta4Integrin**	**1.93E-05**
**TCR**	**NetPath**	**Pathway_TCR**	**2.14E-05**
**Fibroblast growth factor-1**	**NetPath**	**Pathway_Fibroblast__growth__factor-1**	**2.44E-05**
**a6b1 and a6b4 Integrin signaling**	**PID**	**a6b1_a6b4_integrin_pathway**	**2.59E-05**
**BCR**	**NetPath**	**Pathway_BCR**	**6.87E-05**
**EPO signaling**	**INOH**	**None**	**1.02E-04**
**CD4 T cell receptor signaling-NFkB cascade**	**INOH**	**None**	**1.13E-04**
**Leptin**	**NetPath**	**Pathway_Leptin**	**1.23E-04**
**Adrenergic signaling in cardiomyocytes**	**KEGG**	**path:hsa04261**	**1.37E-04**
**Focal adhesion**	**KEGG**	**path:hsa04510**	**1.70E-04**
**EGFR1**	**NetPath**	**Pathway_EGFR1**	**2.30E-04**
**Glioma**	**KEGG**	**path:hsa05214**	**2.35E-04**
**Arrhythmogenic right ventricular cardiomyopathy (ARVC)**	**KEGG**	**path:hsa05412**	**2.51E-04**
**Arrhythmogenic Right Ventricular Cardiomyopathy**	**Wikipathways**	**WP2118**	**2.85E-04**
**Chronic myeloid leukemia**	**KEGG**	**path:hsa05220**	**3.23E-04**
**Human T-cell leukemia virus 1 infection**	**KEGG**	**path:hsa05166**	**3.25E-04**
**Hepatocellular carcinoma**	**KEGG**	**path:hsa05225**	**3.54E-04**
**Hypertrophic cardiomyopathy (HCM)**	**KEGG**	**path:hsa05410**	**4.86E-04**
**TNFalpha**	**NetPath**	**Pathway_TNFalpha**	**5.06E-04**

Table 4: Top 200 genes for colon tumor dataset (D1)

#	Probe ID	Gene Symbol[a]	Gene Full Name[b]
1	X59871	TCF7	transcription factor 7
2	X05276	TPM4	tropomyosin 4
3	X63432	ACTB	actin beta
4	J05032	DARS1	aspartyl-tRNA synthetase 1
5	D26535	DLST	dihydrolipoamide S-succinyltransferase
6	H68220	FAU	FAU ubiquitin like and ribosomal protein S30 fusion
7	T97199	ITGB4	integrin subunit beta 4
8	T56244	PSMB2	proteasome subunit beta 2
9	R16255	PPP3CB	protein phosphatase 3 catalytic subunit beta
10	T70063	EIF4G2	eukaryotic translation initiation factor 4 gamma 2
11	X70040	MST1R	macrophage stimulating 1 receptor
12	U14577	MAP1A	microtubule associated protein 1A
13	U31248	ZNF174	zinc finger protein 174
14	R42029	CACNB3	calcium voltage-gated channel auxiliary subunit beta 3
15	X69295	MSX2	msh homeobox 2
16	R54467	NR2C1	nuclear receptor subfamily 2 group C member 1
17	H19272	AMACR	alpha-methylacyl-CoA racemase
18	T65740	SUB1	SUB1 regulator of transcription
19	T78323	COL4A1	collagen type IV alpha 1 chain
20	H55759	RBP5	retinol binding protein 5
21	H23975	IGHA1	immunoglobulin heavy constant alpha 1
22	X02152	LDHA	lactate dehydrogenase A
23	R52624	HPCA	hippocalcin
24	M63167	AKT1	AKT serine/threonine kinase 1
25	X67235	HHEX	hematopoietically expressed homeobox
26	R07333	ATF4	activating transcription factor 4
27	J05272	IMPDH1	inosine monophosphate dehydrogenase 1
28	T99498	SOST	sclerostin
29	D31883	ABLIM1	actin binding LIM protein 1
30	R70790	AK3	adenylate kinase 3
31	M61858	CYP2C9	cytochrome P450 family 2 subfamily C member 9
32	T61602	RPS11	ribosomal protein S11
33	T86749	CDK4	cyclin dependent kinase 4
34	H40416	ATP5F1B	ATP synthase F1 subunit beta
35	L32977	UQCRFS1	ubiquinol-cytochrome c reductase, Rieske iron-sulfur polypeptide 1
36	X69910	CKAP4	cytoskeleton associated protein 4
37	D25217	MLC1	modulator of VRAC current 1
38	R75893	LPAR6	lysophosphatidic acid receptor 6
39	R71875	GYG2	glycogenin 2

[a] Official symbol provided by HGNC
[b] Official full name description provided by HGNC

40	H09149	ASGR1	asialoglycoprotein receptor 1
41	D63878	SEPTIN2	septin 2
42	X66503	ADSS2	adenylosuccinate synthase 2
43	T88902	MAP3K8	mitogen-activated protein kinase kinase kinase 8
44	T55131	GAPDH	glyceraldehyde-3-phosphate dehydrogenase
45	D28124	NBL1	NBL1, DAN family BMP antagonist
46	T69425	A2M	alpha-2-macroglobulin
47	X66365	CDK6	cyclin dependent kinase 6
48	M26481	EPCAM	epithelial cell adhesion molecule
49	T92259	PSMA6	proteasome subunit alpha 6
50	J04794	AKR1A1	aldo-keto reductase family 1 member A1
51	L13738	TNK2	tyrosine kinase non receptor 2
52	U17899	CLNS1A	chloride nucleotide-sensitive channel 1A
53	M38258	RARG	retinoic acid receptor gamma
54	H65223	AKR7A2	aldo-keto reductase family 7 member A2
55	T61333	TIMP3	TIMP metallopeptidase inhibitor 3
56	R98410	NPC2	NPC intracellular cholesterol transporter 2
57	M84490	MAPK3	mitogen-activated protein kinase 3
58	H07899	VEGFC	vascular endothelial growth factor C
59	X57019	AXL	AXL receptor tyrosine kinase
60	L38810	PSMC5	proteasome 26S subunit, ATPase 5
61	X79066	ZFP36L1	ZFP36 ring finger protein like 1
62	H80240	CTNNA3	catenin alpha 3
63	M35252	TSPAN8	tetraspanin 8
64	U07664	MNX1	motor neuron and pancreas homeobox 1
65	T53396	RPLP1	ribosomal protein lateral stalk subunit P1
66	X66141	MYL2	myosin light chain 2
67	Z19002	ZBTB16	zinc finger and BTB domain containing 16
68	M28373	APP	amyloid beta precursor protein
69	L40027	GSK3A	glycogen synthase kinase 3 alpha
70	T67897	OVGP1	oviductal glycoprotein 1
71	T87527	GDI2	GDP dissociation inhibitor 2
72	H51196	MAPKAPK2	MAPK activated protein kinase 2
73	M88108	KHDRBS1	KH RNA binding domain containing, signal transduction associated 1
74	X68148	SHC1	SHC adaptor protein 1
75	T47584	RPL22P1	ribosomal protein L22 pseudogene 1
76	D16227	HPCAL1	hippocalcin like 1
77	X68277	DUSP1	dual specificity phosphatase 1
78	U03865	ADRA1B	adrenoceptor alpha 1B
79	X03674	G6PD	glucose-6-phosphate dehydrogenase
80	D14663	PSMD6	proteasome 26S subunit, non-ATPase 6
81	X07384	GLI1	GLI family zinc finger 1
82	R77780	B3GNT3	UDP-GlcNAc:betaGal beta-1,3-N-acetylglucosaminyltransferase 3
83	H40699	NXPH3	neurexophilin 3
84	T56604	TUBB	tubulin beta class I
85	L41559	PCBD1	pterin-4 alpha-carbinolamine dehydratase 1
86	H50623	HLA-DRB1	major histocompatibility complex, class II, DR beta 1
87	X57346	YWHAB	tyrosine 3-monooxygenase/tryptophan 5-monooxygenase activation protein beta

88	M11799	HLA-B	major histocompatibility complex, class I, B
89	U13991	TAF10	TATA-box binding protein associated factor 10
90	Y00062	PTPRC	protein tyrosine phosphatase receptor type C
91	L13939	AP1B1	adaptor related protein complex 1 subunit beta 1
92	X80230	CDK9	cyclin dependent kinase 9
93	T89164	RNF181	ring finger protein 181
94	R28608	MAPRE2	microtubule associated protein RP/EB family member 2
95	U19796	MRPL28	mitochondrial ribosomal protein L28
96	M31013	MYH9	myosin heavy chain 9
97	M35878	IGFBP3	insulin like growth factor binding protein 3
98	U05875	IFNGR2	interferon gamma receptor 2
99	J03040	SPARC	secreted protein acidic and cysteine rich
100	R43913	KARS1	lysyl-tRNA synthetase 1
101	T83361	CXCL9	C-X-C motif chemokine ligand 9
102	H26419	APOD	apolipoprotein D
103	H69834	KNG1	Kininogen 1
104	T57882	MYH9	myosin heavy chain 9
105	R73660	IFI30	IFI30 lysosomal thiol reductase
106	T62864	GNAS	GNAS complex locus
107	D11086	IL2RG	interleukin 2 receptor subunit gamma
108	T92736	AP2M1	adaptor related protein complex 2 subunit mu 1
109	X62153	MCM3	minichromosome maintenance complex component 3
110	J05158	CPN2	carboxypeptidase N subunit 2
111	T81919	CREBBP	CREB binding protein
112	J02645	EIF2S1	eukaryotic translation initiation factor 2 subunit alpha
113	T62856	RPL5	ribosomal protein L5
114	R42501	IMPDH2	inosine monophosphate dehydrogenase 2
115	M26383	CXCL8	C-X-C motif chemokine ligand 8
116	L41067	NFATC3	nuclear factor of activated T cells 3
117	T40578	CALD1	Caldesmon 1
118	H78819	CAPS	calcyphosine
119	T54276	PSMB8	proteasome subunit beta 8
120	X02157	EPO	erythropoietin
121	L19437	TALDO1	Transaldolase 1
122	L19760	SNAP25	synaptosome associated protein 25
123	H66786	SULT1E1	sulfotransferase family 1E member 1
124	H75867	SERPINA1	serpin family A member 1
125	H53092	DYRK4	dual specificity tyrosine phosphorylation regulated kinase 4
126	T51250	COX8A	cytochrome c oxidase subunit 8A
127	H41017	CKMT1A	creatine kinase, mitochondrial 1A
128	T63499	HLA-A	major histocompatibility complex, class I, A
129	R98842	PTMA	prothymosin alpha
130	R38736	SEC13	SEC13 homolog, nuclear pore and COPII coat complex component
131	H22939	GYG1	Glycogenin 1
132	T57619	RPS6	ribosomal protein S6
133	M84721	AMPD3	adenosine monophosphate deaminase 3
134	L22214	ADORA1	adenosine A1 receptor
135	M26683	CCL2	C-C motif chemokine ligand 2

136	J03824	UROS	uroporphyrinogen III synthase
137	T85247	COX6C	cytochrome c oxidase subunit 6C
138	T60318	DDX39B	DExD-box helicase 39B
139	R80855	NFKBIA	NFKB inhibitor alpha
140	Z47087	SKP1	S-phase kinase associated protein 1
141	M62831	IER2	immediate early response 2
142	R76825	RANBP1	RAN binding protein 1
143	H28704	SYT7	Synaptotagmin 7
144	X72632	NR1D1	nuclear receptor subfamily 1 group D member 1
145	T72889	IFITM3	interferon induced transmembrane protein 3
146	X12496	GYPC	glycophorin C (Gerbich blood group)
147	M35531	FUT1	fucosyltransferase 1 (H blood group)
148	U09587	GARS	glycyl-tRNA synthetase
149	X13482	SNRPA1	small nuclear ribonucleoprotein polypeptide A'
150	R99916	KIF13A	kinesin family member 13A
151	M27635	HLA-DRB1	major histocompatibility complex, class II, DR beta 1
152	T86444	PPP1R9B	protein phosphatase 1 regulatory subunit 9B
153	H70425	RNU6-998P	RNA, U6 small nuclear 998, pseudogene
154	R44057	PPP2R3B	protein phosphatase 2 regulatory subunit B''beta
155	H29546	NTSR1	neurotensin receptor 1
156	X69392	RPL26	ribosomal protein L26
157	T61627	APOE	apolipoprotein E
158	M28219	LDLR	low density lipoprotein receptor
159	L28809	YBX1	Y-box binding protein 1
160	M10065	APOE	apolipoprotein E
161	M55265	CSNK2A1	casein kinase 2 alpha 1
162	H69695	GLI2	GLI family zinc finger 2
163	J00146	DHFRP3	dihydrofolate reductase pseudogene 3
164	T61609	RPSA	ribosomal protein SA
165	R99907	IRF2	interferon regulatory factor 2
166	R35885	STAG1	stromal antigen 1
167	M95678	PLCB2	phospholipase C beta 2
168	M28827	CD1C	CD1c molecule
169	X17097	PSG4	pregnancy specific beta-1-glycoprotein 4
170	X65024	XPC	XPC complex subunit, DNA damage recognition and repair factor
171	X14968	PRKAR2A	protein kinase cAMP-dependent type II regulatory subunit alpha
172	M77693	SAT1	spermidine/spermine N1-acetyltransferase 1
173	J04102	ETS2	ETS proto-oncogene 2, transcription factor
174	X74295	ITGA7	integrin subunit alpha 7
175	R93337	SERINC2	serine incorporator 2
176	T60437	PMPCB	peptidase, mitochondrial processing beta subunit
177	R85479	APBB3	amyloid beta precursor protein binding family B member 3
178	D00763	PSMA4	proteasome subunit alpha 4
179	U29171	CSNK1D	casein kinase 1 delta
180	T93589	TUBA1C	tubulin alpha 1c
181	R75843	EIF2S3	eukaryotic translation initiation factor 2 subunit gamma
182	R74066	PRKD2	protein kinase D2
183	H08393	WDR77	WD repeat domain 77

184	T57079	FCGR1B	Fc fragment of IgG receptor Ib
185	L27476	TJP2	tight junction protein 2
186	H87193	Pcbp2	poly(rC) binding protein 2
187	H49515	SRP68	signal recognition particle 68
188	T48041	B2M	beta-2-microglobulin
189	D16111	PEBP1	phosphatidylethanolamine binding protein 1
190	X58521	NUP62	Nucleoporin 62
191	U21909	CFL1	cofilin 1
192	T50077	TMEM250	transmembrane protein 250
193	X63071	SON	SON DNA binding protein
194	X86693	SPARCL1	SPARC like 1
195	L34774	OPCML	opioid binding protein/cell adhesion molecule like
196	M14603	MB	myoglobin
197	X59131	USPL1	ubiquitin specific peptidase like 1
198	R23889	TFRC	transferrin receptor
199	R60195	EIF4B	eukaryotic translation initiation factor 4B
200	H63361	ATG10	autophagy related 10

Table 5: Classification accuracy (informedness) of various feature selection algorithms for binary datasets based on three classifiers

Dataset	Top Genes	Classifiers	KLD	GR	SU	RELIEF	SVM-RFE	RSGSA
D1	50	Linear SVM	0.52	0.62	0.63	0.67	0.75	0.86
		RF	0.17	0.58	0.63	0.59	0.54	0.82
		KNN	0.35	0.59	0.63	0.64	0.65	0.84
	100	Linear SVM	0.67	0.68	0.67	0.64	0.75	0.85
		RF	0.21	0.51	0.58	0.55	0.57	0.81
		KNN	0.38	0.58	0.65	0.62	0.63	0.86
	150	Linear SVM	0.62	0.67	0.66	0.62	0.74	0.84
		RF	0.18	0.47	0.48	0.57	0.43	0.83
		KNN	0.39	0.59	0.62	0.60	0.60	0.87
	200	Linear SVM	0.61	0.68	0.66	0.62	0.74	0.86
		RF	0.19	0.46	0.52	0.57	0.50	0.80
		KNN	0.38	0.60	0.63	0.61	0.61	0.84
D2	50	Linear SVM	0.05	0.06	0.14	0.33	0.56	0.74
		RF	-0.01	0.13	0.10	0.17	0.24	0.74
		KNN	0.14	0.14	0.21	0.32	0.60	0.60
	100	Linear SVM	0.01	0.17	0.21	0.34	0.59	0.79
		RF	0.02	0.08	0.10	0.14	0.26	0.74
		KNN	0.07	0.08	0.15	0.29	0.61	0.62
	150	Linear SVM	0.11	0.27	0.19	0.38	0.63	0.79
		RF	0.02	0.12	0.10	0.14	0.16	0.70
		KNN	0.12	0.14	0.18	0.28	0.63	0.59
	200	Linear SVM	0.16	0.33	0.18	0.36	0.68	0.84
		RF	0.00	0.12	0.03	0.10	0.14	0.73
		KNN	0.09	0.20	0.18	0.30	0.59	0.56
D3	50	Linear SVM	0.81	0.90	0.92	0.93	0.96	0.99
		RF	0.61	0.93	0.90	0.80	0.92	0.97
		KNN	0.66	0.85	0.85	0.83	0.90	0.98
	100	Linear SVM	0.83	0.92	0.95	0.92	0.98	0.99
		RF	0.46	0.91	0.87	0.82	0.91	0.98
		KNN	0.65	0.84	0.85	0.79	0.93	0.98
	150	Linear SVM	0.88	0.92	0.92	0.91	0.98	0.99
		RF	0.46	0.91	0.86	0.79	0.89	0.97
		KNN	0.64	0.85	0.84	0.77	0.92	0.99
	200	Linear SVM	0.88	0.93	0.93	0.94	0.97	0.99

		**RF**	0.40	0.88	0.85	0.74	0.89	0.98
		**KNN**	0.64	0.86	0.85	0.79	0.93	0.98
**D4**	**50**	**Linear SVM**	0.26	0.23	0.38	0.45	0.55	0.47
		**RF**	0.25	0.27	0.45	0.37	0.45	0.47
		**KNN**	0.20	0.08	0.37	0.33	0.48	0.43
	**100**	**Linear SVM**	0.31	0.22	0.39	0.40	0.57	0.45
		**RF**	0.30	0.26	0.48	0.33	0.45	0.44
		**KNN**	0.21	0.13	0.31	0.39	0.49	0.43
	**150**	**Linear SVM**	0.25	0.20	0.38	0.44	0.61	0.50
		**RF**	0.24	0.30	0.44	0.37	0.36	0.45
		**KNN**	0.24	0.09	0.34	0.38	0.48	0.41
	**200**	**Linear SVM**	0.29	0.25	0.40	0.43	0.59	0.52
		**RF**	0.24	0.23	0.42	0.34	0.42	0.44
		**KNN**	0.22	0.10	0.35	0.39	0.47	0.46
**D5**	**50**	**Linear SVM**	0.99	0.99	0.99	0.99	1.00	1.00
		**RF**	0.95	0.95	0.96	0.95	1.00	0.99
		**KNN**	0.97	0.98	0.98	0.96	1.00	1.00
	**100**	**Linear SVM**	1.00	1.00	1.00	0.99	1.00	1.00
		**RF**	0.94	0.95	0.94	0.95	1.00	0.99
		**KNN**	0.95	0.97	0.96	0.96	1.00	1.00
	**150**	**Linear SVM**	1.00	1.00	1.00	1.00	1.00	1.00
		**RF**	0.95	0.95	0.94	0.95	1.00	0.99
		**KNN**	0.94	0.96	0.95	0.96	1.00	1.00
	**200**	**Linear SVM**	0.99	1.00	1.00	1.00	1.00	1.00
		**RF**	0.94	0.95	0.94	0.95	0.99	0.99
		**KNN**	0.94	0.96	0.95	0.95	0.99	0.99
**Dataset**	**Top Genes**	**Classifiers**	**KLD**	**GR**	**SU**	**RELIEF**	**SVM-RFE**	**RSGSA**
**Average**	**50**	**Linear SVM**	0.53	0.56	0.61	0.67	0.76	0.81
		**RF**	0.39	0.57	0.61	0.58	0.63	0.80
		**KNN**	0.46	0.53	0.61	0.62	0.73	0.77
	**100**	**Linear SVM**	0.56	0.60	0.64	0.66	0.78	0.82
		**RF**	0.39	0.54	0.59	0.56	0.64	0.79
		**KNN**	0.45	0.52	0.58	0.61	0.73	0.78
	**150**	**Linear SVM**	0.57	0.61	0.63	0.67	0.79	0.82
		**RF**	0.37	0.55	0.56	0.56	0.57	0.79
		**KNN**	0.47	0.53	0.59	0.60	0.73	0.77
	**200**	**Linear SVM**	0.59	0.64	0.63	0.67	0.80	0.84
		**RF**	0.35	0.53	0.55	0.54	0.59	0.79

		**KNN**	0.45	0.54	0.59	0.61	0.72	0.77
**Gain of RSGSA Over other algorithms**	**50**	**Linear SVM**	0.54	0.45	0.33	0.20	0.06	
		**RF**	1.03	0.40	0.31	0.39	0.27	
		**KNN**	0.66	0.46	0.27	0.25	0.06	
	**100**	**Linear SVM**	0.45	0.36	0.27	0.24	0.05	
		**RF**	1.05	0.46	0.33	0.42	0.24	
		**KNN**	0.72	0.50	0.33	0.28	0.06	
	**150**	**Linear SVM**	0.44	0.35	0.31	0.23	0.04	
		**RF**	1.13	0.43	0.40	0.40	0.39	
		**KNN**	0.66	0.47	0.32	0.29	0.06	
	**200**	**Linear SVM**	0.44	0.32	0.33	0.26	0.06	
		**RF**	1.23	0.49	0.43	0.46	0.34	
		**KNN**	0.69	0.41	0.29	0.26	0.07	

Table 6: Classification accuracy (informedness) of various feature selection algorithms for multi-class datasets based on three classifiers

Dataset	Top Genes	Classifiers	SU	GR	KLD	RELIEF	SVM-RFE	RSGSA
D6	50	Linear SVM	0.93	0.94	0.87	0.94	0.96	0.98
		RF	0.92	0.93	0.81	0.91	0.93	0.98
		KNN	0.93	0.89	0.84	0.90	0.95	0.98
	100	Linear SVM	0.94	0.94	0.90	0.95	0.96	0.99
		RF	0.90	0.90	0.71	0.91	0.90	0.98
		KNN	0.92	0.90	0.81	0.94	0.96	0.98
	150	Linear SVM	0.94	0.94	0.90	0.96	0.96	0.99
		RF	0.88	0.89	0.68	0.90	0.90	0.98
		KNN	0.93	0.88	0.81	0.92	0.96	0.98
	200	Linear SVM	0.94	0.94	0.92	0.95	0.96	0.99
		RF	0.87	0.89	0.66	0.88	0.89	0.98
		KNN	0.91	0.89	0.78	0.93	0.96	0.99
D7	50	Linear SVM	0.90	0.90	0.82	0.84	0.92	0.99
		RF	0.84	0.84	0.74	0.75	0.83	0.99
		KNN	0.84	0.80	0.78	0.78	0.89	0.99
	100	Linear SVM	0.90	0.91	0.89	0.86	0.94	0.99
		RF	0.82	0.84	0.64	0.76	0.81	0.99
		KNN	0.84	0.82	0.73	0.79	0.89	0.99
	150	Linear SVM	0.90	0.91	0.90	0.87	0.95	0.99
		RF	0.78	0.82	0.65	0.74	0.78	0.98
		KNN	0.83	0.84	0.76	0.80	0.91	0.99
	200	Linear SVM	0.90	0.91	0.90	0.88	0.94	0.99
		RF	0.77	0.80	0.62	0.73	0.77	0.99
		KNN	0.82	0.83	0.76	0.76	0.91	0.99
D8	50	Linear SVM	0.89	0.91	0.89	0.90	0.95	1.00
		RF	0.81	0.79	0.83	0.82	0.85	0.99
		KNN	0.90	0.90	0.90	0.89	0.94	0.99
	100	Linear SVM	0.91	0.91	0.91	0.91	0.96	1.00
		RF	0.81	0.82	0.81	0.83	0.83	0.99
		KNN	0.90	0.91	0.90	0.91	0.95	0.99
	150	Linear SVM	0.92	0.91	0.93	0.93	0.96	1.00
		RF	0.81	0.84	0.80	0.83	0.82	0.99
		KNN	0.93	0.91	0.90	0.91	0.95	0.99
	200	Linear SVM	0.93	0.92	0.93	0.93	0.96	1.00
		RF	0.82	0.82	0.78	0.82	0.82	0.99
		KNN	0.92	0.91	0.89	0.92	0.95	0.99
D9	50	Linear SVM	0.92	0.93	0.91	0.93	0.98	0.97
		RF	0.87	0.95	0.90	0.91	0.95	0.97
		KNN	0.86	0.87	0.86	0.88	0.98	0.97
	100	Linear SVM	0.94	0.94	0.93	0.94	0.98	0.98
		RF	0.89	0.94	0.83	0.92	0.97	0.97

		KNN	0.89	0.90	0.85	0.91	0.97	0.98
	150	Linear SVM	0.95	0.95	0.93	0.94	0.98	0.98
		RF	0.89	0.93	0.80	0.90	0.97	0.97
		KNN	0.89	0.92	0.83	0.89	0.98	0.97
	200	Linear SVM	0.96	0.95	0.93	0.94	0.98	0.99
		RF	0.85	0.93	0.77	0.93	0.95	0.99
		KNN	0.89	0.89	0.83	0.90	0.97	0.98
D10	50	Linear SVM	0.99	1.00	0.98	0.98	0.99	1.00
		RF	0.98	0.98	0.94	0.97	0.98	1.00
		KNN	0.98	0.99	0.90	0.96	0.98	0.99
	100	Linear SVM	1.00	1.00	0.99	1.00	0.99	1.00
		RF	0.97	0.99	0.89	0.97	0.97	0.99
		KNN	0.98	0.99	0.93	0.97	0.99	1.00
	150	Linear SVM	1.00	1.00	1.00	1.00	1.00	1.00
		RF	0.95	0.99	0.86	0.97	0.96	0.99
		KNN	0.98	1.00	0.92	0.97	0.99	1.00
	200	Linear SVM	1.00	1.00	1.00	1.00	1.00	1.00
		RF	0.97	0.97	0.86	0.96	0.97	0.99
		KNN	0.98	0.99	0.90	0.97	0.99	1.00
Average	50	Linear SVM	0.93	0.94	0.89	0.92	0.96	0.99
		RF	0.88	0.90	0.84	0.87	0.91	0.99
		KNN	0.90	0.89	0.86	0.88	0.95	0.98
	100	Linear SVM	0.94	0.94	0.92	0.93	0.97	0.99
		RF	0.88	0.90	0.78	0.88	0.90	0.98
		KNN	0.91	0.90	0.84	0.90	0.95	0.99
	150	Linear SVM	0.94	0.94	0.93	0.94	0.97	0.99
		RF	0.86	0.89	0.76	0.87	0.89	0.98
		KNN	0.91	0.91	0.84	0.90	0.96	0.99
	200	Linear SVM	0.95	0.94	0.94	0.94	0.97	0.99
		RF	0.86	0.88	0.74	0.86	0.88	0.99
		KNN	0.90	0.90	0.83	0.90	0.96	0.99
Gain of RSGSA Over other algorithms	50	Linear SVM	0.06	0.05	0.11	0.08	0.03	
		RF	0.12	0.10	0.18	0.14	0.09	
		KNN	0.09	0.10	0.14	0.11	0.04	
	100	Linear SVM	0.05	0.05	0.08	0.06	0.03	
		RF	0.11	0.09	0.26	0.11	0.10	
		KNN	0.09	0.10	0.18	0.10	0.04	
	150	Linear SVM	0.05	0.05	0.06	0.05	0.02	
		RF	0.14	0.10	0.29	0.13	0.11	
		KNN	0.09	0.09	0.18	0.10	0.03	
	200	Linear SVM	0.04	0.05	0.05	0.05	0.03	
		RF	0.15	0.12	0.34	0.15	0.12	
		KNN	0.10	0.10	0.19	0.10	0.04	
